# On the Injurious Effects of Mercury in the Treatment of Disease

**Published:** 1860-07

**Authors:** 


ART. IV.—*On the Injurious Effects of Mercury in the Treatment of Disease.* By S. O. HABERSHON, M.D. Churchill: London, 1860. pp. 86.

IF there is any error of which the medical profession has been peculiarly guilty, it is the too frequent and indiscriminate use of mercurial preparations in the treatment of disease. Precisely because these articles are among our most powerful and trustworthy remedies—because when administered some effect can surely be looked for—they have been resorted to until, as we firmly believe, their *abuse* is more frequent than their *use.* We write of what we have seen; our field of observation has been the Western States—a region where, from the prevalence of malarious diseases, some forms of mercury are peculiarly called for, and where their beneficial effects, as cathartics, have encouraged and sustained a resort to them. If our statement requires proof, we can point to it in the popular voice and action of the region of which we speak: the regular physician is known too generally as a "*calomel doctor,*" and a tribe of shamefully ignorant quacks, under the name of "eclectics," are thriving upon the popular prejudice against all other than botanical remedies.

We had hoped that this error was confined to our own country, and to this section of it, but we learn from a perusal of Dr. Habershon's book that it is committed quite as frequently in England, with all her hospitals for clinical instruction, and with those legal restrictions against men assuming the responsibilities of practice without due qualification, which we have not. According to our author's statements, mercury is abused in England as much as it can be anywhere, and quite sufficiently to justify the reproaches of French and German physicians upon the partiality of British practitioners for this heroic remedy.

"For more than half a century the preparations of mercury have been employed in the manner suggested by Dr. Hamilton, and at length it is used with most unsparing hand; our text-books recommend mercury in almost every disease, and the inexperienced unheedingly follow the dictum: in acute and in chronic disease, in so-called inflammatory and non-inflammatory ailment, in abnormal conditions of the nervous, respiratory, circulatory, or digestive systems, in renal or cutaneous disease, in blood diseases or in injuries, mercury finds its advocates, and has been given as if it were the panacea of human disease."

"We find with some persons that, whatever the ailment, however produced, mercury is given; 'antiphlogistics' administered to prevent injury or to cure. Thus, a man receives a mechanical injury; he is mercurialized, as we have known, to prevent so-called inflammation taking place; there is headache, sometimes because the heart cannot propel the blood into the brain, (for there may be intense headache from exhaustion as well as repletion,) mercury is given; or blood gravitates into the uterine, rectal, or other veins, mercury is used to unload them, although the condition may arise from exhaustion; a patient is affected with *pneumonia*, it may be from injury, mercury is the remedy; from mechanical retardation of blood, mercury is given; from pressure on the pneumogastric and consequent changed nervous supply, again mercury is given, as if the changes were the same in each instance, whatever the disturbing cause."

It is very evident, then, that a work upon the injurious effects of mercury was much needed by the profession, and had the author confined himself to showing the needlessness of the remedy in many of the pathological conditions he has mentioned, and to pointing out the injury it is sure to inflict when administered in cases to which it is not adapted, we should have welcomed his work with unmingled pleasure. The errors above alluded to, however, are errors of individuals, not of the science; that patients are sometimes mercurialized after an injury, "to prevent so-called inflammation taking place," we do not doubt; but certainly they are not so treated by any well-informed physicians, nor in accordance with any of the received doctrines of pathology. But Dr. Habershon does not thus limit himself to a consideration of the evils arising from mercury ignorantly or carelessly given; he claims that it not only fails of exerting any beneficial effect, but is actually injurious in a class of diseases for which heretofore it has been considered one of our most powerful and most reliable remedies. There is no doctrine of medical science more constantly acted upon in practice, or which is better supported by authority, than that mercury exerts a great influence in promoting the resolution of inflammation, in checking inflammatory effusion, and in hastening the absorption of solid and liquid exudations. This doctrine the author not only doubts, but denies; he maintains that mercury has no power of controlling the inflammatory process, that it exerts no influence upon the absorption of exudations, and that the lowering effect upon the system, which it is well known to produce, tends to increase instead of to diminish effusion. Now the abandonment of the views held by the profession for those presented by the author, amounts to a revolution in practice,—there is no escaping this conclusion,—and we place the question in this light not because it throws any evidence into the scale, but to show that it behooves him who writes and those who read to understand fully the importance of the subject under discussion, to carefully examine the arguments advanced and the cases detailed, and only to make a decision after thorough investigation and calm reflection.

Those who have read the "Clinical Lectures" of Dr. Bennett, and those who are acquainted with the writings of the late Dr. Todd, will at once see that the author of the work before us is not alone in his views in regard to the injurious effect of mercury when administered in acute inflammations, and its want of power either to control this morbid process or to effect the removal of its products. He has placed himself by the side of men eminent for their learning, and occupying high positions in the profession, who not only doubt the power of mercury, but boldly challenge the truth of many points of theory, and the efficacy of many measures of treatment, which are sanctioned by authority, and daily acted upon by the mass of the profession. These men maintain that venesection, as a remedy for inflammation, is not only never beneficial, but always injurious; that disease presents no such distinctions as "sthenic" and "asthenic;" that there is no such thing as a "change of type" of disease; that the constitution of man does not undergo modifications at different periods of time; and that the whole course of treatment termed "antiphlogistic," and of which the administration of mercury is but a part, exerts a deleterious effect upon the patient, and tends more to his destruction than to the cure of the disease under which he suffers.

It will be seen, then, that we cannot enter here upon the discussion of the question as to the injurious or beneficial effects of mercury when administered for acute inflammation, the only point in the work upon which we should take issue with the author, and the only point to which we cannot give an assent. It is but a small part of a much wider and more important question, which covers the whole treatment of inflammation, and leads to an examination of the most important parts of general pathology. Intimately connected as it is with points which must first be examined and decided, we feel compelled to leave it for another occasion, and limit ourselves here to presenting to our readers some information in regard to the contents of the book under consideration.

The first chapter of this work considers "the general effects of mercury," and every one will admit that to ascertain fully and to state fairly the physiological action of a remedy, be it new or old, should precede an inquiry into its action upon disease. We do not find anything new in this chapter, while we think that far too much space is occupied with details of the poisonous effects of mercury when given to the extent of producing salivation. To dwell so much at length in this chapter, which should be a plain statement of physiological facts, upon the abuse of the remedy, and to depict so vividly the horrors of salivation, which is not a result of its judicious use, suggests to a candid mind the inquiries whether the author is not writing under the influence of prejudice, and whether the view he takes of the subject is not obscured by partiality and prejudice.

The second chapter is on "the mode of action of mercury," and opens with quotations from various writers showing the different theories of the manner in which the preparations of this metal produce their effects. We admit that widely different explanations are given, but this is of no consequence unless it is also shown that the same authors disagree as to the classes of disease in which mercurials can be beneficially exhibited; this they do not, being as unanimous in this respect as they are discordant as to whether the remedy is a special stimulant, entropic, tonic, sedative, or catalyptic. A "spanœmic" it certainly is; "alterative" is the favorite term with the profession, and although the author thinks it but a term for our ignorance, which is undoubtedly true, he does not give us any more definite explanation of its mode of action.

"We must confess that we are not satisfied with any of the theories of mercurial action; and the facts known do not warrant us to state more than that after its introduction into the system in small medicinal doses, the circulating fluid is changed in character, nutritive processes modified or checked, and glandular organs excited to increased action."

This chapter contains a suggestion to use the terms "metatrophy" to denote the phenomena of changed nutrition in ordinary acute and chronic inflammation, and "paratrophy" for other forms of perverted nutrition. We should have no objection to the adoption of these terms did we think they were necessary, and could agree with the author, who believes that the *term* inflammation is the cause of a great amount of malpractice. That this word is a vestige of the days of ignorance we admit; that its original meaning influences practice now we do not believe.

The third chapter treats "of special diseases in which mercury is injurious," and we can but copy the list. Let the reader bear distinctly in mind, however, that the author alludes to the injurious effect of the constitutional influence of mercury, and would not abolish its use in these diseases if used as a cathartic, or in occasional small doses, not continued long enough to affect the general system.

"1. In strumous diseases of the brain, lungs, abdomen, skin, bones, etc.

"2. In degenerative changes of advanced life, as atheromatous deposit in the vessels, leading to apoplexy, ramollissement of the brain, etc., in fatty degenerations of the heart, aneurism.

"3. In fevers and exanthems, as scarlet fever, typhus, measles, small-pox.

"4. In conditions consequent on exhaustion, whether from over-fatigue, preternatural drain, mental anxiety, insufficient food, loss of blood.

"5. In passive congestions of the lungs, uterus, etc., in states of weakness and loss of power.

"6. In cancerous diseases.

"7. In degeneration of the kidneys.

"8. In diseases of the mucous membranes it is of very questionable utility, as bronchitis, enteritis, cæcal disease, dysentery, etc.

"9. In diseases called inflammatory, as of the membranes and substance of the brain, of the lungs and pleura, of the pericardium and peritoneum, in croup, etc.; while in some cases the products of disease become absorbed and health restored, in very many instances the malnutrition consequent on the mercury leads to increased effusions.

"10. In rheumatism and its complications the advantage is not equivalent to the injurious effect."

We assent to the first seven of these propositions without hesitation; although the first is directly opposed to the advice of so high authority as Dr. Graves, in his Clinical Lectures, we think the profession generally will agree with the author in believing that the constitutional effect of mercury is always injurious in strumous disease. We regret that the eighth proposition is not treated at greater length; inflammatory diseases of the alimentary canal constitute a large proportion of cases which come under the care of the physician in this country in the summer months, mercury is largely used in their treatment, and often, we have no doubt, with injurious effects. Had the author pointed out carefully the circumstances indicating their administration, or those forms of disease or conditions of system in which they would be likely to prove deleterious, he would have conferred a very great favor upon the profession. The rich stores of experience gathered by his countrymen in India, and other tropical climates, might furnish facts which he has been unable to gather at home.

The doctrines of the ninth and tenth propositions are the most important part of the book, as we have before shown; to these statements in regard to the injurious effect of mercury in inflammations we cannot assent; neither, as we have already said, can we enter at length into the consideration of them, because they are most intimately connected with other questions which must be previously investigated. All we can say here is, that they are in direct opposition to the recorded experience of the profession, and all authorities upon the action of medicines. We prefer, therefore, to render the good old Scotch verdict of "not proven," and await the result of further investigation, which time will surely bring us.

Each of the propositions quoted above is made the subject of some remarks in the work, and illustrative cases are reported. Some of these cases are excellent; we would especially mention the one of strumous peritonitis, which contains a striking illustration of the value of opium in this disease. The largest number of cases are of pneumonia, a disease which seems a favorite one with the teachers of the new doctrines in regard to the evil effects of venesection, mercury, and antiphlogistics generally. We believe there is a good reason for this; a reason which should never be lost sight of by the practitioner in perusing their writings. We allude to the widely-changed hygienic conditions under which the patients in these cases are placed, in the hospital where they are treated, compared with the situations in which they contracted the disease, and the circumstances by which they were surrounded. The poorly-clad, half-starved being is taken from the streets, or from some filthy, over-crowded lodging in a dark court or alley, and, after a warm bath, is placed in a clean bed, and breathes the warm air of a well-lighted and well-ventilated ward, is supplied with palatable food, and his wants are ministered to by experienced nurses. All this has a powerful effect upon him; these means alone tend strongly to restore him to health, and have an especial influence in promoting the subsidence of such a disease as pneumonia. The change is certainly sufficient to interfere seriously with any deductions which might be attempted as to the action of remedies; and we cannot consent to abandon old and tried modes of treatment for new ones, which are supported only by the result of cases observed in the wards of a metropolitan hospital.

The "conditions in which the administration of mercury is advantageous," forms the subject of the fourth and last chapter of the work, and we must copy the list to represent the author fairly, but have no space for comment:—

"1. In retained excretions, as from the bowels, liver, etc., it is often of great value.

"2. As a speedy and efficient purgative.

"3. As an excitant to the excretory organs in some forms of dropsy, of embarrassed heart, of bronchitis, etc.

"4. In small or occasional doses where its more free or continued use would be detrimental, in some of the classes to which we referred to its administration as being injurious.

"5. In syphilitic diseases.

"6. In some forms of gastrodynia and irritability of stomach, the symptoms are relieved by it.

"7. In Asiatic cholera, although most severe cases have recovered after its administration, it is of doubtful efficacy.

"8. Its local application in some cutaneous diseases, and chronic ulceration, is certainly salutary."

Since we cannot speak in terms of unqualified praise of Dr. Habershon's work, let us, in concluding our notice of it, state distinctly the extent to which we think he merits it, and the points upon which we think he has fallen short of doing all he might have done. We think he has failed to present, as fully as he ought, the injurious effects of mercury in cases where, from some peculiarity of the disease, or of the constitution of the patient, its action is different from the general rule. Syphilis, for instance, and especially as it occurs in strumous patients, furnishes such a subject, and one upon which a chapter of far more value than any in the work could be written; the remedy is abused in the treatment of syphilis as much probably as in any other disease, and it is not easy, if possible, to secure its beneficial effect in strumous subjects without injury to the constitution of the patient.

We think that in omitting to estimate the influence of great changes in the hygienic conditions of the cases he reports, he has not given that due attention to all the circumstances bearing upon the course of the disease which we have a right to expect in a clinical teacher. We think, too, that the work is not as complete as it should be, in view of the important change its doctrines would effect, if adopted, in the practice of medicine. We cannot look upon a work of this character as we should upon one designed only as a modest contribution to doctrines already held; it is antagonistic and revolutionary in its character, at issue with the received teachings of pathology, and we have a right to ask for a thoroughness and completeness which we should not demand under other circumstances.

On the other hand, so far as the author points out the folly of having recourse to mercurials in every case of disease, impresses the criminality of pursuing a routine practice with such powerful remedies, and presents proof of their injurious effects when needlessly given, he deserves our most hearty thanks, and we most cheerfully accord them. So far as this portion of the work is concerned, we wish it were in the hands of every young physician in the country; if its perusal but caused him to pause and give another moment's reflection as he extended his hand toward the mercurial preparations of his pocket-case, or took up his pen to write a prescription, it would be of great service to himself, and of incalculable benefit to his patients.

If any one is disposed to think, from the remarks we have made upon the work, that we hold Dr. Habershon censurable for the attempt he has made to show that mercurials exert an injurious influence upon the inflammatory process, let us hasten to remove the false impression. Far be it from us to maintain that the present state of medical science is so perfect as to be incapable of improvement, and its doctrines so well established as to demand no re-examination. On the contrary, we know that the rapid advances made in chemistry, minute anatomy, physiology, and pathology, make it imperative upon us to investigate from time to time the principles of our practice, and see that our means of curing disease keep pace with other branches of the science. We cannot assent to his propositions, because he has not presented proof enough to enforce conviction of their truth, and because his testimony conflicts with that of other observers equally competent. But neither will we deny them; there is a state of mind termed by Göthe "*thätige skepsis*," which we recommend to every young man as he investigates the truth of Dr. Habershon's propositions, and we recommend to him not only an *active*, but an *intelligent skepticism*—a skepticism which understands all doctrines, examines all evidence, scrutinizes all facts, and is open to conviction when a sufficient amount of truth shall be presented. Let him observe carefully his cases and the effects of every remedy he administers; let him try to attribute to the right source every change which occurs in the patient for better or for worse, and be satisfied with nothing short of a complete understanding of every case he treats. If the author of this work, and those who hold similar views, fail in establishing the truth of the novel doctrines they have announced, the ground upon which we stand will be firmer under our feet; if, on the contrary, they succeed in proving them correct, we are convinced that to range ourselves by their side we must advance, for it is not in the nature of the human mind to retrograde. We commend, therefore, the subject-matter of the work to the consideration of the profession, hoping it will receive that attention and examination which its importance deserves.

